# Pegylated liposomal mitomycin C prodrug enhances tolerance of mitomycin C: a phase 1 study in advanced solid tumor patients

**DOI:** 10.1002/cam4.491

**Published:** 2015-07-14

**Authors:** Talia Golan, Tal Grenader, Patricia Ohana, Yasmine Amitay, Hilary Shmeeda, Ninh M La-Beck, Esther Tahover, Raanan Berger, Alberto A Gabizon

**Affiliations:** 1 Sheba Medical CenterTel HaShomer, Israel; 2 Shaare Zedek Medical CenterJerusalem, Israel; 3 Lipomedix Pharmaceuticals Ltd.Jerusalem, Israel; 4 Texas Tech University Health Sciences Center-School of PharmacyAbilene, Texas; 5 Hebrew University-School of MedicineJerusalem, Israel

**Keywords:** Clinical trial, liposome, mitomycin C, prodrug

## Abstract

Mitomycin C (MMC) has potent cytotoxicity but cumulative toxicity limits widespread use. In animals, pegylated liposomal mitomycin C lipid-based prodrug (PL-MLP) was well tolerated and more effective than free MMC. We evaluated PL-MLP in patients with advanced cancer. Twenty-seven patients were treated in escalating dose cohorts of 0.5–3.5 mg/kg (equivalent to 0.15–1.03 mg/kg MMC) every 4 weeks for up to 12 cycles, unless disease progression or unacceptable toxicity occurred. Pharmacokinetics were assessed during cycles 1 and 3. Per protocol maximum tolerated dose was not reached at 3.5 mg/kg. However, prolonged thrombocytopenia developed after repeated doses of 3 mg/kg or cumulative doses of 10–12 mg/kg. Dose-related grade 3 or higher adverse events included fatigue, anemia, and decreased platelets. *C*_max_ and AUC_0-∞_ increased linearly over the dose range 0.5–2.0 mg/kg, and greater than linearly from 2.5 to 3.5 mg/kg; there were no significant differences in clearance of MLP between cycles 1 and 3. Median *t*_1/2_ was 23 h among dose cohorts, with no trend by dose or cycle. One patient had a partial response. Stable disease was observed in 10 patients across all dose levels. PL-MLP has a long circulation time, was well tolerated, and can be administered to heavily pretreated patients at a single dose of 3.0 mg/kg and cumulative dose of 10–12 mg/kg before development of prolonged thrombocytopenia; this is nearly threefold the equivalent dose of MMC tolerated historically. This formulation may be active in a variety of tumor types and is better tolerated than free MMC.

## Introduction

Mitomycin C, an antitumor antibiotic, was discovered and first tested in the 1950's 1; it was approved for clinical use in the US in 1974. It has been used in treatment of a variety of solid tumors, including bladder, breast, cervical, colon, gastric, non-small cell lung, and pancreatic cancer 2. Its mechanism of action involves alkylation and cross-linking of DNA after a bioreductive activating step 3,4. Due to cumulative toxicities, including thrombocytopenia, hemolytic-uremic syndrome, and interstitial pulmonary fibrosis, systemic use of mitomycin has fallen out of favor in the past decades, with the exception of its use with curative intent in anal cancer in conjunction with radiotherapy and 5-FU 5. Nevertheless, because mitomycin C is generally not associated with multidrug resistance through common mechanisms 6,7, it could be a useful agent if toxicity were reduced.

Pegylated liposomes have been used to improve the pharmacokinetics and, thereby, pharmacodynamics of another important anticancer agent, doxorubicin 8, leading to a formulation in broad clinical use 9. Pegylated liposomes have an extended blood circulation lifetime as compared to traditional liposomes lacking the polymer coating, allowing systemically administered liposomal drug to reach a target region, cell, or tissue more efficiently. Due to the enhanced permeability and retention effect, pegylated liposomes tend to accumulate in tumors and in sites of inflammation 10. Preclinical studies of a pegylated liposomal mitomycin C lipid-based prodrug, 2, 3-(distearoyloxy)propane-1-dithio-4'-benzyloxycarbonyl-MMC (PL-MLP) demonstrated prolonged circulation and an improved therapeutic index as compared to free mitomycin C (MMC) in several tumor models tested 11. The prodrug was designed to intercalate in the lipid layer as a lipid component. It is cleaved by ubiquitously reducing agents abundant in tumors, particularly the thioredoxin-thioreductase system 12, releasing active mitomycin C locally. The levels of free mitomycin C in the plasma are negligible, indicating that the prodrug remains successfully entrapped in circulating liposomes, enabling targeting of tumor and various tissues, while decreasing systemic exposure.

In this first-in-man study, we determined the dose-limiting toxicities (DLTs) and maximum tolerated dose (MTD) of the pegylated liposomal MMC prodrug. We also studied the pharmacokinetics of PL-MLP and observed patients for evidence of antitumor activity.

## Patients and Methods

### Patient selection

Patients aged 18–80 with inoperable, recurrent, or metastatic malignant solid tumors who had failed prior therapy or for whom no standard therapy was available were candidates for the study. Patients had to have ECOG performance status ≤ 2, and adequate organ function, as delineated in the protocol. Patients with NYHA class IV heart failure, LVEF ≤ 40%, COPD ≥ stage 3, serum albumin <30 g/L, cirrhosis, pregnancy or lactation, or who were currently undergoing other antineoplastic treatment were excluded.

The protocol was approved by the ethics committee at each participating institution and performed in accordance with the International Conference on Harmonization Good Clinical Practice guidelines, and the Declaration of Helsinki. The trial was also registered at clinical trials.gov with identifier NCT01705002. All patients signed informed consent.

### Treatment regimen

PL-MLP, provided by Lipomedix Pharmaceuticals Ltd, Jerusalem, Israel, under the trade name of Promitil®, was manufactured by Northern Lipids Inc. (Burnaby, BC). The dose of PL-MLP is measured on the basis of its MLP content, the active pharmaceutical ingredient of the product. PL-MLP is dispensed as an aqueous suspension of liposomes in vials of 5 or 10 mL containing 5 mg MLP per mL. The prescribed dose was diluted in 250 mL 0.9% saline for administration. Initial cohorts of three patients each were to be treated at each dose level, 0.5, 1.0, 1.5, 2.0, 3.0, and 3.5 mg/kg, infused over 2 h. The infusion protocol required initially a slow drip rate which was raised at stepwise increments until reaching the target of 2 mg/min. If patients manifested signs of acute infusion reaction, the infusion was to be stopped, and after a brief period resumed at a lower rate and then re-escalated gradually to the target rate. For a 70 kg patient, the starting dose would be 35 mg, which contains 10.3 mg of mitomycin C-equivalents (3.4 mg of MLP contains 1 mg of MMC). This is equivalent to about 6 mg/m^2^ MMC for an average patient with 1.72 m^2^ BSA. Dosing was on a weight rather than body surface area basis, as is often done for drugs which primarily disperse in the intravascular space, such as liposomes and antibodies, given the well-established correlation between body weight and blood volume 13. If two patients at a given dose level developed dose-limiting toxicities, dose escalation was to be stopped. If one patient at a given dose level developed a dose-limiting toxicity, three additional patients were to be treated at that level. If no further patients developed dose-limiting toxicity, escalation could proceed. However, if one or more of the second group of three patients also developed dose-limiting toxicity, escalation was to be stopped.

Dose-limiting toxicity was defined as follows:

- Nonhematologic toxicity: any toxicity ≥ grade 3, other than grade 3 nausea and vomiting, fever, or hepatic toxicity which recovered to grade 1 prior to the scheduled time for the next treatment cycle.
- Hematologic toxicity:
  - Grade 4 neutropenia, anemia, or thrombocytopenia ≥7 days' duration, or
  - Toxicity during the first three cycles of grades 2 through 4 and which fails to resolve to grade 1 within 52 days of the last treatment of Promitil (28 days of cycle +24 days' delay).

Patients who did not experience dose-limiting toxicity during the first cycle could continue on treatment once every 4 weeks. The long 6-week interval of mitomycin C was felt to be inappropriate for PL-MLP because of the diminished toxicity of the prodrug in preclinical studies 14 and its use as single agent in this study. The dose interval of 4 weeks was selected based on the clinical experience with pegylated liposomal doxorubicin, also a long-circulating liposome, which is given every 4 weeks 8,9. As mitomycin C is known to have cumulative toxicity, patients were followed carefully during subsequent treatment cycles. If two or more patients receiving a given dose were to demonstrate dose-limiting toxicity during cycle 2 or 3, the dose level below that at which dose-limiting toxicity developed would be considered the repeated treatment maximum tolerated dose.

### Patient assessment

Patients were examined, questioned regarding adverse events and use of concomitant medications, and underwent routine safety laboratory testing periodically throughout the study. Tumors were assessed at baseline and every 12 weeks on study using RECIST 1.1 criteria applied to imaging appropriate for the individual patient's tumor. Tumor response was based on investigator assessment.

### Pharmacokinetic assays

Pharmacokinetic assessments of MLP were performed at cycles 1 and 3. PK analysis of MLP was performed on ≥2 mL blood collected in K-EDTA coded prelabeled tubes collected prior to infusion and at the following time points postend of infusion: 15 min, 30 min, 1 h, 2 h, 4 h, 10 ± 1 h, 24 h ± 1, 72 h ± 1, 7 days ±1, 14 days ± 1, 21 days ± 1, and 28 days ± 1. Samples were refrigerated immediately upon collection. Plasma was separated by centrifugation within 2 h, and then frozen at −20°C. Pharmacokinetic analyses were performed at the Laboratory of Experimental Oncology at the Shaare Zedek Medical Center.

For sample preparation, plasma samples were diluted ×10 in isopropanol (IPA), followed by centrifugation for thorough extraction of MLP and protein precipitation. The analytical technique was RP-HPLC with UV detection at 360 nm using an HPLC instrument, Merck Hitachi LaChrom, and a column, Phenomenex Hypersil BDS 5u C18 130A (150 × 4.60 mm, 5 micron). The mobile phase was methanol: IPA 70:30. For pharmacokinetic analysis, the sensitivity lower limit of this quality-controlled assay was established at 1 *μ*g/mL plasma.

The pharmacokinetic characterization of MLP included the following parameters: *C*_max_, AUC_0-∞_, MRT, *t*_1/2_, Cl, and *V*_D_. The pharmacokinetic parameters were calculated with SAS^©^ version 9 or higher software (SAS, Cary, NC, USA) using noncompartmental methods for analysis of plasma concentrations of MLP. To calculate pharmacokinetic parameters, values below the limit of quantitation (BLQ) and missing samples were ignored in the pharmacokinetic analysis.

### Complement activation

Complement activation during PL-MLP infusion was assessed by measuring the soluble terminal complement complex (Sc5b-9) in the preinfusion and first postinfusion plasma sample for the first cycle with an ELISA kit (MicroVue Complement SC5b-9 Plus EIA, Quidel Co., San Diego, CA) similar to previously described methods 15.

## Results

### Study population

A total of 27 patients were entered into the study. Demographic variables and tumor types are shown in Table1. The most common tumor type was colorectal carcinoma, followed by ovarian and bladder carcinoma. All but two patients were ECOG performance status 0 or 1 at screening. All patients had received at least one prior regimen of cytotoxic chemotherapy for advanced disease, and most had received multiple lines of chemotherapy. In addition, about half of them had received biologic agents, mainly monoclonal antibodies appropriate for their tumor type.

**Table 1:** Patient demographics and tumor characteristics

Parameter	
Number of patients	27
Male/female	11/16
Age, median (range), years	66.1 (42–78)
ECOG performance status: median (range)	1 (0–2)
0	13 (48%)
1	12 (44%)
2	2 (7%)
Tumor types, *N* (%)	
Colorectal	11 (41%)
Ovarian or primary peritoneal	4 (15%)
Bladder	3 (11%)
Other^1^	9 (33%)

1 One patient each: breast, cervix, cholangiocarcinoma, gall bladder, hypopharynx, melanoma, pancreas, stomach, unknown origin.

### Treatment administered and patient disposition

Table2 shows treatment administered and patient disposition by initial dose level. A total of 103 cycles of treatment were administered, for a mean of 3.8 (median = 3) cycles per patient. Six patients were entered at 2.0 mg/kg as an initial expansion cohort before further escalation, and six were entered at 3.5 mg/kg as a confirmatory expanded cohort of the highest planned dose after protocol revision. Seven patients received extended treatment of PL-MLP beyond the third cycle, 13 received the planned minimum three cycles, and seven patients did not reach cycle 3, six due to early progression or clinical deterioration and one due to an infusion reaction in cycle 2.

**Table 2:** Treatment administered and patient disposition

Dose level	0.5	1.0	1.5	2.0	2.5	3.0	3.5
*N*	3	3	3	6	3	3	6
Number of cycles - median (range)	11 (3–12)	3 (2–8)	3 (3)	2.5 (2–8)	3 (3–5)	3 (2–4)	3 (1–4)
Reason for discontinuation							
Tumor progression^1^	1	1	3	6	3	0	4
Adverse event (not treatment-related)	0	1	0	0	0	0	1
Treatment-related adverse event	1	1	0	0	0	3	1
Other	1^2^	0	0	0	0	0	0

1 Either radiologically, by tumor markers or clinically.

2 Completed 12 cycles. Discontinued with stable disease, no dose-limiting toxicity.

During the extended treatment phase, after the first three cycles, the dose of PL-MLP was increased to 1.5 mg/kg for two patients in the 0.5 mg/kg cohort and one patient in the 1.0 mg/kg cohort at cycle 8.

Across all dose groups, 18 patients (67%) discontinued treatment for either radiologic or clinically assessed progressive disease; two patients (7%) due to adverse events assessed as unrelated to study medication, and 6 (22%) due to adverse events assessed as related to study medication. One patient in the 0.5 mg/kg cohort discontinued after 12 cycles without dose-limiting toxicity or progressive disease. Five patients discontinued due to thrombocytopenia: one each at 0.5 mg/kg (cycle 11), 1.0 mg/kg (cycle 8), and 3.5 mg/kg (cycle 4); and two at 3.0 mg/kg (cycles 3 and 4). One patient had a moderate hypersensitivity reaction during the second infusion, which was terminated prematurely.

The planned dose intensity per dose cohort was maintained relatively well along the study except for the highest dose cohort of 3.5 mg/kg (see Table S1).

### Adverse events

All but one patient reported one or more adverse events; 59% reported grade 3 or worse adverse events. Twenty-three patients (85%) reported adverse events at least possibly related to study medication, most of which were mild or moderate; 26% reported grade 3 or worse adverse events at least possibly related to study medication. The incidence of grade 3 or higher adverse events, regardless of causation, increased with increasing dose: 7/15 patients (47%) in the 0.5–2.0 mg/kg dose groups; 4/6 patients (67%) in the 2.5 and 3.0 mg/kg dose groups; and, 5/6 patients (83%) in the 3.5 mg/kg dose group. The incidence of grade 3 or higher adverse events considered at least possibly related to study medication was higher in the 3.5 mg/kg dose group (3/6, 50%) than across all lower dose groups (4/21, 19%). The incidence of adverse events (AE) by dose group at all cycles is summarized in Table S2.

Adverse events experienced by at least one-third of patients, regardless of causation or grade, were fatigue (44%), thrombocytopenia (41%), decreased appetite (33%), and vomiting (33%). Among these, only the decrease in platelets was dose-dependent. At doses of 0.5–2.5 mg/kg, 6/18 (33%) of patients had thrombocytopenia, and one (5.6%) had a grade 3 decrease. At doses of 3.0 or 3.5 mg/kg, 5/9 (56%) of patients had thrombocytopenia of any grade, and 3/9 (33%) had a grade 3 or 4 decrease. A list of the most frequent adverse events reported as related or possibly related to treatment is presented in Table3. Thrombocytopenia and fatigue were the most common ones.

**Table 3:** Adverse events reported as related or possible related to study treatment in ≥3 patients

Adverse events	No. events (No. subjects)							
	*N* patients = 27; Dose range = 0.5–3.5 mg/kg							
Dose (mg/kg)	0.5	1.0	1.5	2.0	2.5	3.0	3.5	All
*N* patients	*N* = 3	*N* = 3	*N* = 3	*N* = 6	*N* = 3	*N* = 3	*N* = 6	*N* = 27
Nonhematological								
Nausea	1 (1)			1 (1)	3 (3)			5 (5)
Vomiting			1 (1)	3 (1)	1 (1)		1 (1)	6 (4)
Decreased appetite	1 (1)		1 (1)	1 (1)	2 (2)		1 (1)	6 (6)
Asthenia			1 (1)		1 (1)		1 (1)	3 (3)
Fatigue				1 (1)	1 (1)	5 (3)	3 (1)	10 (6)
Infusion-related reaction				1 (1)		1 (1)	1 (1)	3 (3)
Hot flush				1 (1)			2 (2)	3 (3)
Hematological								
Anemia	2 (1)			1 (1)	3 (2)		2 (1)	8 (5)
Low platelets	3 (2)	2 (1)		1 (1)	1 (1)	7 (3)	5 (2)	19 (10)

Grade 3 or higher adverse events experienced by at least 10% of patients were fatigue, increased gamma glutamyl transferase (GGT) and thrombocytopenia (15% each), and anemia (11%). Anemia, fatigue, and thrombocytopenia occurred mainly at the higher dose levels. The grade 3 increases in GGT were not considered treatment-related, but rather disease-related in all cases.

Twelve patients (44%) developed serious adverse events (SAE) during the study. Two of these patients died due to the SAE; in neither case was this considered related to study medication. One patient in the 0.5 mg/kg group developed increased bilirubin and hepatobiliary disorder and died 54 days after the third dose of study medication. The death was attributed to tumor progression based on liver biopsy. One patient in the 3.5 mg/kg group developed dyspnea and a lung infection and was hospitalized 3 weeks after the third and last dose of study medication. The patient deteriorated clinically and died 6 weeks after the last dose of study medication. One patient of the 0.5 mg/kg dose cohort developed a serious adverse event, pulmonary embolism, considered possibly related to study medication after 12 cycles of study medication. The patient recovered from the event, but did not receive additional study medication after the SAE. No patient developed hemolytic-uremic syndrome or significant pulmonary toxicity attributed to study medication. Prior oncologic therapies and cancer diagnoses of patients who had SAE's are presented in Table S3.

### Determination of maximum tolerated dose

No patient developed a dose-limiting toxicity. Thus, per the definition in the protocol, the MTD was not reached. However, as several patients developed prolonged thrombocytopenia after several cycles of therapy across the higher dose levels, particularly at 3 mg/kg, the dose was not escalated beyond 3.5 mg/kg. Therefore, 3 mg/kg was considered the maximal single recommended dose.

**Figure 1:**
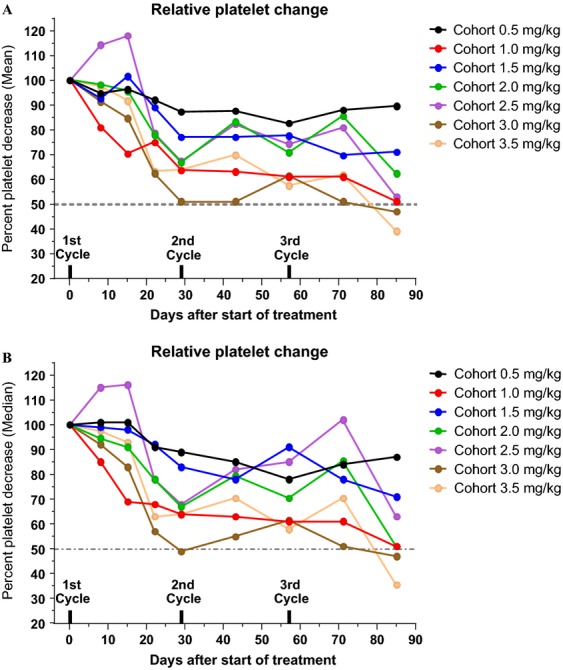
Relative (%) change in platelet counts from pretreatment count (normalized to 100) across the various dose levels and along the first three cycles. (A) Mean % change; (B) Median % change. Note the downward trend for both mean and median values. The 3.0 and 3.5 mg/kg dose cohorts fall below 50% after the third cycle. Mean and Median values followed a similar pattern. For clarity, SEM values were not plotted.

**Figure 2:**
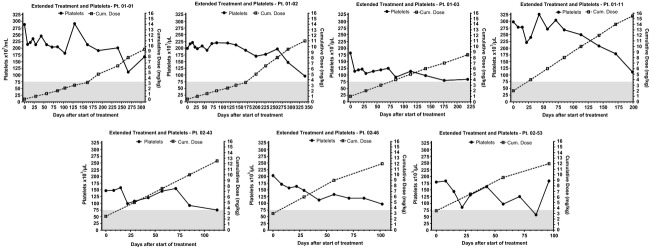
Platelet Counts as a function of cumulative dose of PL-MLP in seven patients receiving extended treatment (beyond three cycles). Gray area marks Grade 2–4 toxicity zone for low platelets. Note the downward trend of platelet counts as cumulative dose increases.

The relationship between dose cohort level and relative (percent) drop in platelet count along the first three treatment cycles is shown in Figure1. The detailed profile of platelet counts per patient and per dose cohort during the first three treatment cycles is presented in Figure S1. A trend to lower platelet counts with increasing dose and increasing number of cycles can be noticed. The median cumulative dose of MLP administered to patients who discontinued therapy due to thrombocytopenia was 9.5 mg/kg (range 8.5–14 mg/kg). In contrast, the median cumulative dose for patients who discontinued due to other reasons, primarily tumor progression, was 4.5 mg/kg (range 1.5–15.5 mg/kg). In the group of seven patients who received extended treatment (4 to 12 cycles), the median cumulative dose was 12 mg/kg (range 9.5–15.5 mg/mg) as seen in Figure2. Based on all these observations, the maximum tolerable cumulative dose in this heavily pretreated patient population appears to be 10–12 mg/kg. The tolerable cumulative dose did not appear to vary depending on individual dose level: patients in the lower dose and higher dose treatment groups developed significant platelet count decrease in the same range of cumulative dose.

### Pharmacokinetics

Total plasma MLP concentrations were measured. Prior studies had shown that plasma MLP is present in plasma only in liposomal form, as it is insoluble in water and does not bind to plasma proteins 14, implying that the levels of total MLP are identical to the levels of liposomal MLP. In plasma stability studies, PL-MLP is a very stable formulation with no evidence of MLP loss from liposomes 14. However, if liposomal MLP is thiolytically cleaved by thiol donors (whether small molecules or proteins), it generates free MMC which rapidly leaks out from the liposomes 16.

**Figure 3:**
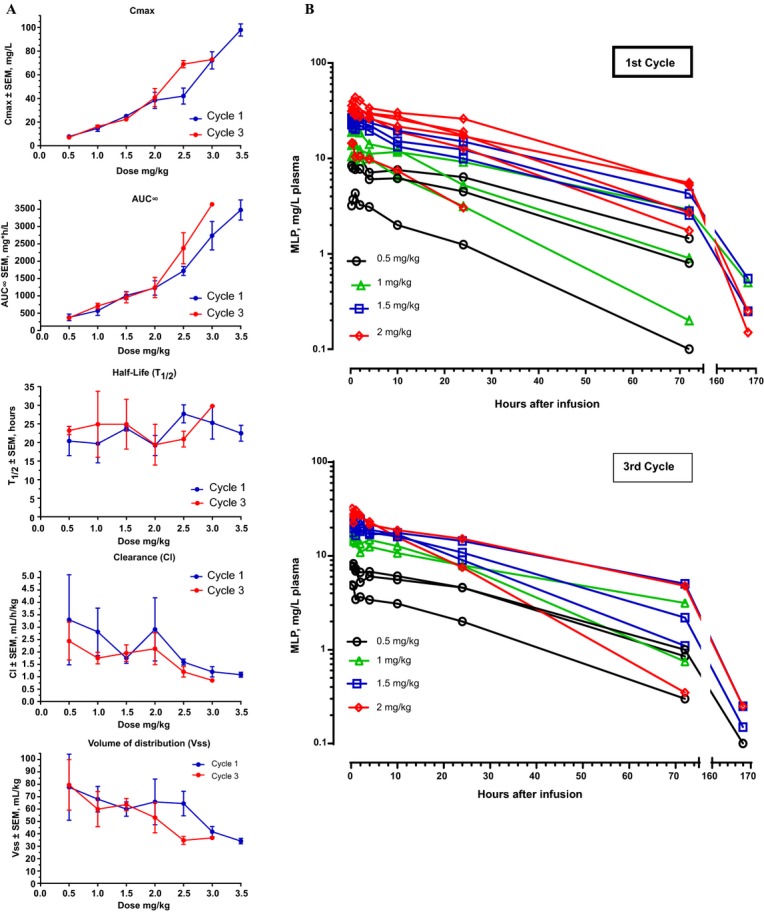
Pharmacokinetics of PL-MLP. (A) Changes in PK parameters as a function of dose level. Note the nonlinearity in Cmax and AUC for dose levels is >2 mg/kg. There was no perceptible change in *t*_1/2_ with dose, but there was a slight reduction in clearance and volume of distribution with increasing dose. The third cycle of Cohort 3.5 mg/kg was given at a dose of 3 mg/kg and was therefore not plotted. (B) Clearance of MLP following PL-MLP administration to dose cohorts 0.5–2 mg/kg. Note the monophasic exponential clearance in the first 72 h. There was no noticeable difference in the clearance of MLP between the first and third cycles. Each color line represents an individual. Values of MLP < 1 mg/L were plotted but not used for analysis of PK parameters.

Mean values of relevant pharmacokinetic parameters are presented in Table4. Plasma peak levels of MLP were much higher in all patients than those reported for free mitomycin C, as expected from a stable pegylated liposomal formulation with distribution restricted to the intravascular compartment. The change in the mean values of key pharmacokinetic parameters as a function of dose is depicted in Figure3A. *C*_max_ and AUC_0-inf_ increased linearly over the dose range 0.5–2.0 mg/kg. From 2.5 to 3.5 mg/kg, the increase was greater than linear suggesting some degree of clearance saturation. Mean *C*_max_ at 3.0 mg/kg was 71.3 mg/L at cycle 1, and 81.2 mg/L at cycle 3; each of these was twice the respective *C*_max_ at 2.0 mg/kg despite the fact that the increase in dose was only 150%. Likewise, mean AUC_0-∞_ at 3.0 mg/kg was 2541 mg*h/L at cycle 1 and 3079 mg*h/L at cycle 3, which is 2.7 and 3.4 times, respectively, the AUC_0-inf_ values at a dose of 2.0 mg/kg.

**Table 4:** Pharmacokinetic parameters of PL-MLP

Treatment group, mg/kg		0.5	1.0	1.5	2.0	2.5	3.0	3.5
	Cycle							
*N*	1	3	3	3	6	3	3	6
*N*	3	3	2	3	3	3	2	2^1^
*C*_max_, mg/LMean (SD)	1	7.7 (3.2)	14.7 (4.5)	25.1 (2.8)	38.4 (16.7)	42.0 (11.8)	72.1 (12.7)	89.0 (24.1)
	3	7.0 (1.8)	16.2 (1.9)	22.3 (2.2)	40.7 (13.2)	69.0 (5.2)	81.7 (12.7)	59.2 (3.6)^1^
AUC_0-∞_ mg^*^h/LMean (SD)	1	254 (167)	442 (238)	882 (195)	1209 (635)	1591 (224)	2397 (548)	2794 (627)
	3	240 (97)	581 (110)	826 (270)	1112 (504)	2246 (774)	3009 (634)	1663 (141)^1^
*t*_½_, hMean (SD)	1	20.4 (6.8)	19.7 (9.0)	23.8 (1.2)	19.9 (6.3)	27.7 (4.2)	25.3 (7.6)	22.9 (4.4)
	3	23.2 (1.9)	24.9 (12.6)	24.9 (11.6)	19.4 (9.5)	20.9 (3.7)	23.8 (8.4)	21.6 (2.9)^1^
CL, L/hMean (SD)	1	0.22 (0.15)	0.22 (0.15)	0.12 (0.02)	0.19 (0.19)	0.12 (0.02)	0.10 (0.03)	0.08 (0.05)
	3	0.17 (0.04)	0.13 (0.05)	0.13 (0.02)	0.14 (0.06)	0.09 (0.03)	0.07 (0.02)	0.10 (0.02)^1^
*V*_D_, LMean (SD)	1	5.4 (1.8)	5.2 (1.8)	4.3 (0.5)	4.6 (2.7)	4.8 (1.6)	3.3 (0.4)	2.8 (1.7)
	3	5.65 (1.0)	4.2 (0.6)	4.8 (1.6)	3.8 (1.7)	2.5 (0.5)	2.5 (0.4)	2.9 (0.1)^1^

1 The third cycle of Cohort 3.5 mg/kg was given at a dose of 3 mg/kg.

Plasma clearance of MLP followed a straight monoexponential curve during the first 1 to 3 days after infusion (Fig.3B), as typically expected from a stable, nonleaky, liposomal drug preparation with first-order clearance kinetics.

Terminal half-life (*t*_1/2_) varied from 18 to 27 h, with no trend by dose or cycle. The mean *t*_1/2_ was 22.5 h in the first cycle and 22.4 h in the third cycle for the entire patient group. The median intercohort *t*_1/2_ was 23 h for both cycle 1 and cycle 3.

Free MMC levels were measured by LC-MS in five randomly selected patients at dose levels 0.5–2.0 mg/kg, and found to be below the limit of quantification (2 ng/mL) at all time points (data not shown). It was, therefore, decided not to continue the analysis of plasma samples for the presence of MMC.

### Tumor response

**Figure 4:**
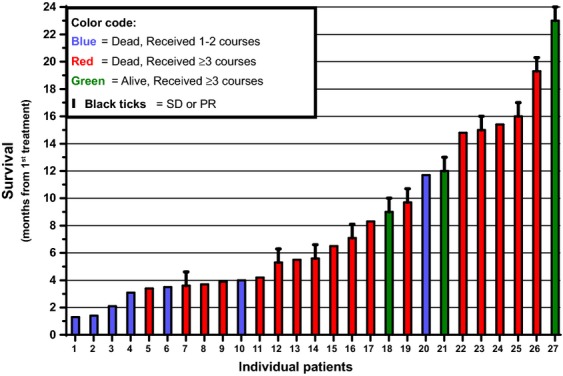
Survival of all 27 PL-MLP-treated patients entered to the study. Records from start of study (Nov 2012) to last data check (Nov 2014). Median survival of all patients (*N* = 27) and of responding (black ticks, SD + PR) patients (*N* = 11) was 5.6 and 9.7 months, respectively.

At data cutoff for the present analysis (24 months after start of study), 24 of the 27 patients entered to the study had died. Median survival was 5.6 months for all patients (95% CI: 3.7–9.7 months). Survival per individual patient is shown in Figure4. As expected, patients who completed the first three study cycles were more likely to achieve stable disease and survive longer. The median survival of responding patients was 9.7 months (*n* = 11).

One patient, with cervical carcinoma treated with 3.5 mg/kg, had a partial response observed at cycle 3. A total of 10 patients across all dose levels had stable disease. Patients with stable disease were distributed across the dose levels, including all three treated with 0.5 mg/kg, with no apparent dose–response relationship.

**Figure 5:**
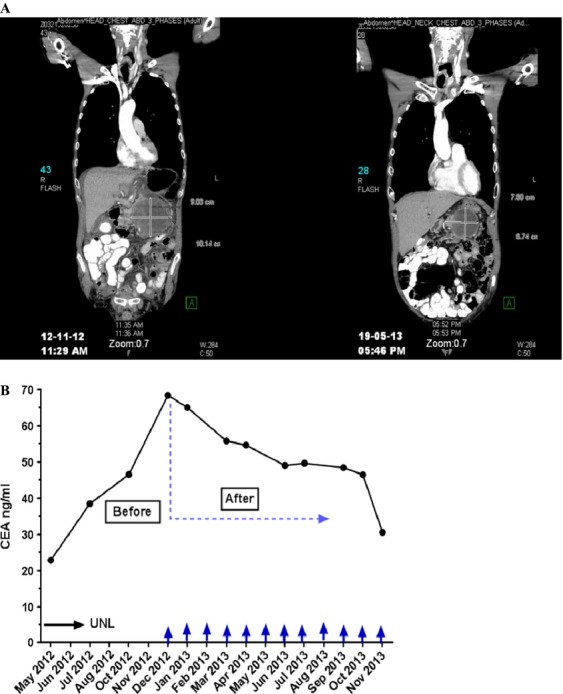
Antitumor activity of PL-MLP. (A) CT image showing response to treatment in a melanoma patient with disappearance of ascites and volume shrinkage of abdominal tumor mass (>30%) for 6 months. Reported as Stable Disease (as two small lesions in lung and forehead—not shown—increased in size). (B) Plot showing CEA decreases in a colon cancer patient receiving 12 cycles of PL-MLP. From start of PL-MLP, the CEA level dropped gradually by >2-fold. Stable Disease in CT-scan for 12+ months.

The patient with melanoma had significant and sustained shrinkage of one intra-abdominal lesion (Fig.5A) but did not qualify as a partial responder due to parallel growth of two other smaller lesions in lung and forehead. Another patient with triple negative breast cancer had clinically significant and sustained improvement of a malignant pleural effusion but could not continue treatment beyond the fourth cycle due to the prolonged thrombocytopenia.

Cancer-related serum tumor markers were followed for several patients. One patient, who had colon carcinoma and was treated with 0.5 mg/kg, had a significant and sustained decrease in CEA and CA-19-9 extending beyond cycle 12 when treatment was discontinued (Fig.5B). Radiologic imaging (CT) showed stable disease during the entire study period. Two additional patients had declines of at least 25% in one or more serum tumor markers at least once during the course of the study, though these were not followed for prolonged periods.

### Complement activation

Complement activation has been described during liposome infusions and appears to mediate the acute infusion reactions known as complement activation-related pseudoallergy (CARPA) 17. There was significant interpatient variability in Sc5b-9 levels, although it was above the upper limit of normal (490 ng/mL; 18) in only four patients. Of these, two had elevated levels at baseline which did not further increase after PL-MLP administration. Sc5b-9 levels in the other two patients increased significantly by 74–108% after PL-MLP infusion and were associated with the development of a transient acute infusion reaction.

## Discussion

Unlike many other chemotherapeutic agents, MMC is usually not associated with multidrug resistance. Early studies demonstrated single-agent activity in a wide range of common tumors 1. In combination therapy with capecitabine, recent studies also show that MMC may be a valuable agent in advanced colorectal cancer in patients failing to irinotecan and 5-FU treatments 19. However, hematological and renal toxicities following repeated treatment with MMC have led clinicians to limit its use 20. Thus, its safety profile is an obstacle toward exploiting its activity against malignancies, in general, and multidrug-resistant tumors, in particular. Severe toxicity and other side effects may be significantly reduced and efficacy enhanced if favorable tissue distribution changes and selective delivery to the tumor site can be achieved. Liposomal drug delivery exploits the fluid transport dynamics of abnormal tumor vessels, which, unlike healthy vessels of normal tissues, allow significant extravasation of circulating liposomes. Drug retention is further enhanced due to lack of effective lymphatic drainage in the tumor tissue 10. The use of long-circulating pegylated liposomes with a prodrug requiring thiolytic and reductive activation to generate an alkylating species was intended to target the drug in a controlled release mode to tumor tissue, which often overexpresses thioredoxin signaling 12,21.

The pharmacokinetics of PL-MLP correlated closely with the animal data. In contrast to the short half-life of free mitomycin C of approximately 15 min 22, the half-life of PL-MLP was approximately 1 day. Pharmacokinetics was linear at doses up to 2.0 mg/kg, but *C*_max_ and AUC_0-∞_ increased disproportionately at doses of 2.5 mg/kg or greater. The *t*_1/2_ was similar across dose levels, and pharmacokinetics was similar at cycles 1 and 3, suggesting no damage to the liposome clearance mechanism. In contrast, retardation of clearance has been observed in patients treated with three cycles of pegylated liposomal doxorubicin 23.

Only one of 18 patients treated at doses of 2.5 mg/kg or lower developed grade 3 or higher thrombocytopenia, as compared to three of nine patients treated with doses of 3.0 or 3.5 mg/kg. Similarly, grade 3 or higher anemia developed in only one of 18 patients treated with doses of 2.5 mg/kg or lower, as compared to two of nine treated with 3.0 or 3.5 mg/kg. Only one patient, treated with 3.5 mg/kg MLP, developed grade 2 neutropenia; no patients developed grade 3 or 4 neutropenia. Thus, myelosuppression on a per cycle basis was clearly dose related.

As with the parent drug, cumulative myelosuppression was dose-limiting. While generally not severe, its persistence prompted discontinuation of treatment. Across all dose groups, the median cumulative dose resulting in discontinuation of therapy due to thrombocytopenia was 10 mg/kg MLP. This is equivalent to a cumulative-free mitomycin dose of approximately 120 mg/m^2^ for a 70-kg/1.72 m^2^ patient, that is, approximately six cycles of 20 mg/m^2^ each. This is at least twice the lifetime maximal cumulative dose of MMC generally tolerated, thus indicating that the liposomal prodrug substantially reduces the toxicity of its active metabolite, MMC, in agreement with preclinical studies 11,14,16.

Most patients had extensive prior chemotherapy. Thus, the cumulative thrombocytopenia noted may represent a worst-case scenario; patients with minimal or no prior chemotherapy may tolerate higher cumulative doses of PL-MLP.

In a rodent study, equivalent doses of PL-MLP and free mitomycin C resulted in similar increases in tumor control 11. However, given that the dose of PL-MLP could be escalated several-fold higher than that of free mitomycin C, PL-MLP resulted in an overall improvement in efficacy over mitomycin C without significant toxicity. In our study, we were able to escalate the equivalent dose of mitomycin C approximately threefold and the dose intensity by 4.5-fold in comparison to what is generally administered with free mitomycin, without significant acute toxicity. The limiting factor was cumulative thrombocytopenia, which occurred at similar total doses of PL-MLP whether given at a low or high dose.

In this study, neutropenia was uncommon and, when it occurred, mild. We did not note the severe but less common side effects of mitomycin C, such as pulmonary toxicity, nephrotoxicity, and hemolytic-uremic syndrome, though their absence may have been a function of the number of patients treated. Transient and self-limiting CARPA reactions occurred in 7.4% (2/27) of patients and do not represent a significant problem with the current mode of administration.

Two of three patients treated with the lowest dose tested, 0.5 mg/kg (equivalent to 6 mg/m^2^ mitomycin C), increased to 1.5 mg/kg (equivalent to 18 mg/m^2^ mitomycin C) from cycle 8, had stable disease for 11 months and >1 year. This suggests that even relatively low doses, which were very well tolerated, may be clinically active. However, except for one short-lived partial response, efficacy was limited to stable disease. Thus, in this study, the antitumor activity of PL-MLP remains difficult to evaluate.

In summary, PL-MLP was well tolerated and could be administered at substantially higher individual and cumulative doses as compared to free MMC. Future studies will demonstrate whether this larger safety window will result in improvement in treatment efficacy.

## Supporting Information

**Table S1.** Administered dose as % of planned dose. **Table S2.** Summary of adverse events per dose cohort. **Table S3.** Description of SAE and patient details (cont'd next page). **Figure S1.** Platelet counts and dose escalation cohorts.
