# Supplementary material for: Pegylated liposomal mitomycin C prodrug enhances tolerance of mitomycin C: a phase 1 study in advanced solid tumor patients
Source: Cancer Med. 2015 Jul 14;4(10):1472–83. doi: 10.1002/cam4.491 (PMC4618618; doi:10.1002/cam4.491)
Supplement: Supplementary file 1 [file cam40004-1472-sd1.docx]

**Supplemental Table S1:** Administered Dose as % of Planned Dose

|  | **Administered Dose as % of Planned Dose (N patients)** | | | | | | |  |
| --- | --- | --- | --- | --- | --- | --- | --- | --- |
| **Dose Cohort (mg/kg)** | **0.5** | **1.0** | **1.5** | **2.0** | **2.5** | **3.0** | **3.5** | |
| **Cycle 1** | **99.5 (3)** | **98.8 (3)** | **100.0 (3)** | **99.9 (6)** | **100.0 (3)** | **99.2 (3)** | **100.0 (6)** | |
| **Cycle 2** | **99.5 (3)** | **98.3 (3)** | **99.9 (3)** | **100.0 (6)** | **100.0 (3)** | **99.9 (3)** | **96.4 (4)** | |
| **Cycle 3** | **100.2 (3)** | **100.0 (2)** | **100.4 (3)** | **100.0 (3)** | **100.0 (3)** | **100.0 (2)** | **92.8 (4)** | |
| **Cycle 4** | **100.0 (2)** | **100.0 (1)** |  | **100.0 (1)** | **100.0 (1)** | **100.3 (1)** | **68.2 (1)** | |
| **Cycle 5** | **100.6 (2)** | **100.0 (1)** |  | **100.0 (1)** | **100.0 (1)** |  |  | |
| **Cycle 6** | **100.6 (2)** | **100.0 (1)** |  | **100.0 (1)** |  |  |  | |
| **Cycle 7** | **100.6 (2)** | **100.0 (1)** |  | **100.0 (1)** |  |  |  | |
| **Cycle 8** | **300.0 (2)** | **150.5 (1)*** |  | **75.0 (1)** |  |  |  | |
| **Cycle 9** | **300.0 (2)*** |  |  |  |  |  |  | |
| **Cycle 10** | **300.0 (2)*** |  |  |  |  |  |  | |
| **Cycle 11** | **300.0 (2)*** |  |  |  |  |  |  | |
| **Cycle 12** | **298.5 (1)*** |  |  |  |  |  |  | |

* The relative dose increase in these patients is due to a change in dose with escalation to 1.5mg/kg from the 8^th^ cycle, after the dose cohort of 1.5 mg.kg was safely cleared..

**Supplemental Table S2.** Summary of Adverse Events per Dose Cohort

| Dose level (mg/kg) | 0.5 | 1.0 | 1.5 | 2.0 | 2.5 | 3.0 | 3.5 |
| --- | --- | --- | --- | --- | --- | --- | --- |
| N patients | 3 | 3 | 3 | 6 | 3 | 3 | 6 |
| Patients with grade 3 or higher AE | 2 | 3 | 0 | 2 | 2 | 2 | 5 |
| Patients with grade 3 or higher ADR* | 1 | 0 | 0 | 1 | 1 | 1 | 3 |
| Patients with SAE | 2 | 2 | 0 | 4 | 0 | 0 | 4 |
| *ADR: adverse drug reaction, i.e., adverse event possibly, probably or certainly related to study medication. | | | | | | | |

**Supplemental Table S3.** Description of SAE and patient details (cont’d next page)

| **Dose** | **Subject** | **Previous lines of therapy*** | **Cancer diagnosis** | **AE term** | **Grade (severity)** | **Relation to study drug** | **Action** |
| --- | --- | --- | --- | --- | --- | --- | --- |
| 0.5 mg/kg | 01-02 | Bevacizumab, Fluorouracil, Capecitabine, Irinotecan, Oxaliplatin, Radiotherapy (pelvic mass) | Colon Cancer | Pulmonary Embolism | Severe | Possible | None |
| 0.5 mg/kg | 02-31 | Gemcitabine, Cisplatin, Paclitaxel, Caboplatin, MVAC ( Methotrexate + Vinblastine + Adriamycin + Cisplatin), Radiotherapy (retroperitoneum) | Bladder Cancer | Hepatobilliary Disorders (Jaundice) | Death | Unlikely related | Study drug discontinued (liver biopsy showed metastatic carcinoma) |
| 1.0 mg/kg | 01-04 | Cyclophosphamide Doxorubicin, Paclitaxel, Docetaxel, Carboplatin, Pegylated Liposomal Doxorubicin, Topotecan, Gemcitabine | Ovarian cancer | Fracture Of Lower Limb Right Hip | Severe | Unrelated (trauma- accident) | None |
| 1.0 mg/kg | 01-05 | Oxaliplatin, Bevacizumab, Irinotecan, Fluorouracil | Colon Cancer | Confusion | Moderate | Unlikely related | None |
| 2.0 mg/kg | 01-09 | Carboplatin, Gemcitabine | Bladder carcinoma | Urinary Tract Infection | Severe | Unrelated | None |
| 2.0 mg/kg | 01-11 | Capecitabine | Colon Cancer | Pleural Effusion | Moderate | Unrelated | None |
| 2.0 mg/kg | 02-35 | Carboplatin, Paclitaxel, Gemcitabine, Topotecan | Ovarian cancer | Ischemic Stroke | Moderate | Unrelated | None |
| 2.0 mg/kg | 02-36 | Gemcitabine, Cisplatin, Paclitaxel, Carboplatin | Urothelial Ca (Unknown origin) | Small Intestinal Obstruction | Moderate | Unlikely related | None |
| 3.5 mg/kg | 02-49 | Bevacizumab, Fluorouracil, Oxaliplatin, Irinotecan, AFLTBco6097 (Study Alfercopt), Cetuximab | Colon cancer | 1. Fatigue  2. Cognitive Disturbance | Severe  Severe | Unlikely related  Unrelated | Subject discontinued from study  None |
| 3.5 mg/kg | 02-52 | Bevacizumab, Fluorouracil, Irinotecan, ,Oxaliplatin Panitumumab | Colon cancer | Lung Infection, Dyspnea | Life-threatening | Unrelated | None |
| 3.5 mg/kg | 02-53 | Bevacizumab, Fluorouracil, Oxaliplatin, Irinotecan | Colon cancer | 1. Vomiting  2. Colon Obstruction  3. Anemia  4. Fever | Mderate  Severe  Severe  Mild | Unrelated  Unrelated  Unrelated  Unrelated | None  Study drug temporarily stopped  None  None |
| 3.5 mg/kg | 02-55 | Oxaliplatin, Capecitabine, Paclitaxel, Fluorouracil, Irinotecan | Stomach cancer | Colon Obstruction | Severe | Unrelated | Subject discontinued from study |

* Fluouracil (5FU) was administered in most cases together with Calcium Folinate (Leucovorin)

**Supplemental Figure S1:** Platelet counts and dose escalation cohorts. Cohort 2 mg/kg is plotted in both 2^nd^ and 3^rd^ panels to facilitate inter-cohort comparison. Grey area marks Grade 2-4 toxicity zone for low platelets. Platelet values exceeding the top of the Y axis are written in full in the plot area. In Cohort 3.5 mg/kg, some of the patients received 3mg/kg in 2^nd^ and 3^rd^ cycles. Each color line represents an individual.

Page Left Intentionally Blank
